# Evaluation of stray radiation to the operator for five hand-held dental X-ray devices

**DOI:** 10.1259/dmfr.20180301

**Published:** 2019-02-09

**Authors:** Richard Smith, Richard Tremblay, Graeme M Wardlaw

**Affiliations:** 1 Consumer and Clinical Radiation Protection Bureau, Health Canada, Canada

**Keywords:** x-rays, radiography, dental equipment

## Abstract

**Objectives:**

Evaluate stray radiation to the operator, as represented by a plane within the significant zone of occupancy (SZO), produced by five models of hand-held intraoral dental X-ray devices (HIDXDs).

**Methods:**

The stray radiation for five models of HIDXDs was measured, using an anthropomorphic tissue-equivalent head phantom as a scattering object. An ionization chamber was used to measure the air kerma (μGy) at 63 positions in a 160 cm high by 60 cm wide plane that was 10 cm behind the X-ray device, identified as being within the SZO.

**Results:**

Based on the measured air kerma from stray radiation of five different HIDXDs, the estimated annual air kerma at all measured spatial positions was calculated. When calculated using a median air kerma of 0.8 mGy at the distal end of the cone, as typically required for digital image receptors,^1^ the ranges for estimated annual air kerma in the SZO across the devices were 0.14–0.77 mGy for the median, 0.41–1.01 mGy for the mean, and 1.32–2.55 mGy for the maximum. Similarly, when calculated using a median air kerma of 1.6 mGy as typically required for D-speed film,^2^ the ranges for estimated annual air kerma across the devices were 0.28–1.54 mGy for the median, 0.83–2.03 mGy for the mean, and 2.64–5.10 mGy for the maximum.

**Conclusions:**

From measured air kerma values of stray radiation in the SZO, estimated annual exposures to the operator for HIDXDs are expected to be greater than from conventional wall-mounted or portable devices activated from a protected area (at a distance or behind shielding). HIDXDs should therefore only be used when patient accessibility makes their use necessary and the use of a portable device on a stand or a wall-mounted device is not reasonably feasible. This approach would keep occupational radiation exposures of dental workers as low as reasonably achievable.

## Introduction

Dental radiography is a valuable tool in the field of dental care, supporting the diagnosis, treatment and management of dental health concerns. Intraoral dental X-ray exposures, where the image receptor is placed inside the mouth of the patient, have traditionally been performed with wall-mounted dental X-ray devices. Such wall-mounted devices allow the operator to stand at a reasonably safe distance from the patient, or behind a wall or structure that provides shielding, during the X-ray exposure. In recent years, hand-held intraoral dental X-ray devices (HIDXDs) have become available, where the operator holds the device while performing an intraoral dental X-ray exposure. While the use of HIDXDs raises some concerns for operator safety, it should be noted that in some situations their use can offer potential benefits over conventional wall-mounted dental X-ray devices with respect to dental care access for patients.^3^

HIDXDs consist of an X-ray tube assembly with the irradiation switch directly on the body of the device. The physical form of the device generally resembles the shape of a large hand-held camera or hair dryer. The device may include a protective shield at the end of the dental cone intended to reduce backscatter radiation to the operator, and this backscatter shield may be removable or permanently affixed. The intended use of hand-held equipment requires that the operator stand beside the patient during the X-ray exposure. The devices are used to produce conventional dental X-ray images using intraoral X-ray image receptors, similar to those produced by wall-mounted X-ray devices.

When using HIDXDs, the close proximity of the operator to both the patient and the X-ray device raises concerns about possible increases of radiation dose to the operator when compared to conventional wall-mounted intraoral dental X-ray devices. Numerous studies have reported a range of stray radiation exposure measurements to the operator from HIDXDs, using various instruments and methodologies.^4–14^ For these studies, the strength of conclusions is limited by aspects of the methodologies or study protocols. Key limiting factors in the methodologies include: use of an inappropriate phantom to represent the patient that could affect the stray radiation field that is measured, as opposed to a tissue-equivalent phantom that would accurately recreate the stray radiation field^6,7,10,11^; use of inappropriate means or detector for measurement of radiation dose given the X-ray imaging technique,^7,11,14^ and; inadequate number of measurement points, which may not adequately characterize the heterogeneous stray radiation field around the operator of a HIDXD.^4–13^ In the referenced previous studies, no more than 12 measurement points were used when the number of points was specified. Limiting factors with regards to radiation detectors include use of a detector with an inadequate response time of greater than 0.25 s which may not reliably measure the air kerma from shorter exposure times of dental X-ray devices which were used in previous studies. Also in regards to radiation detectors, a previous study had a similar methodology as in this study, where a HIDXD was used with a tissue-equivalent phantom as scattering target, and an unspecified number of air kerma measurements were made in a plane perpendicular to the beam direction and through the focal spot of the device.^14^ This previous similar study found no measurable air kerma in the plane through the focal spot, however the detectors used are examples of inappropriate detectors that were not intended by the manufacturer to be used for measurement of stray radiation. More explicitly, a 0.6 cm^3^ farmer ionization chamber and a solid state dosemeter were used to measure scatter radiation, where the ionization chamber volume and capturing cross-section is not conducive to the relatively low X-ray fluence of stray radiation, and the manufacturer stated intended use of the solid state dosemeter is acceptance testing and routine quality control measurements on diagnostic X-ray units, not scatter or leakage measurements.

In this study, the air kerma from the stray radiation field created by five different HIDXDs has been measured using a tissue equivalent phantom, radiation detectors with an appropriate ionization chamber volume and a response time of no greater than 1 ms that are suited for the X-ray timer setting of 1.0 s, and 63 measurement points relevant to the position of the operator to characterize the stray radiation field.

## Methods and Materials

Air kerma measurements were taken for five HIDXDs from different manufacturers as listed in Table 1. Some devices had removable dental cones, and all devices included a back scatter shield for which there was some variation in lead equivalence thickness and diameter of the shield (Table 1). The backscatter shield and removable dental cones were in place for all measurements on each device throughout work presented here.

**Table 1.** Specifications of HIDXDs

Device (Manufacturer)	Fixed tube voltage of HIDXD (kVp)	Fixed tube current of HIDXD (mA)	Manufacturer specified lead equivalence of backscatter shield (mm Pb)	Diameter of backscatter shield (cm)	Manufacturer specified total filtration of HIDXD (mm Al equivalent)	Measured half-value layer of HIDXD (mm Al equivalent)	Measured Air Kerma per current-time product of HIDXD (mGy/mAs)
BIOX-IPX 0 (DigiMed Co., Ltd., Seoul, Korea)	60	3	Not stated	15.5	2.8	2.87	0.887
Nomad Pro2 (Aribex, Inc., Orem, UT)	60	2.5	Not stated	15.5	≥1.5	2.22	1.473
SAF-3000 (Safari Dental Inc., Boisbriand, Québec)	60	2	0.35 @ 70 kVp	14.8	2.3	2.40	1.264
Xray2Go (Osstem Implant Co., Ltd., Geumcheon-gu, Korea)	60	2	Not stated	15.8	1.6	2.76	0.830
Zen-PX2 (Genoray Co., Ltd., Seongnam, Korea)	60	2	Not stated	15.0	1.8	2.76	0.720

HIDXD, hand-held intraoral dental X-ray device.

Summary of select device specifications and X-ray beam quality for the five HIDXDs evaluated for stray radiation. Backscatter shields were in place for all devices. The source-to-skin distance for each device was 20 cm with the removable cones in place. A timer setting of 1.0 sec was used for the measured air kerma output for all devices. For each device, the timer setting is the only adjustable loading factor, thus differences in output for a given device are solely a result of changes to selected exposure time.

Initial measurements of the primary beam X-ray air kerma were made for each device using a timer setting of 1.0 s. Three air kerma measurements were taken at the end of the dental cone of each device, with a source-to-skin distance of 20 cm. Further measurements were made for each device to assess the half-value layer, tube voltage accuracy, timer accuracy, and linearity of air kerma. Measurements were made with an Unfors R/F MAM detector (Model 8202031 J, Unfors RaySafe, Inc., Billdal, Sweden) and Unfors multimeter (8201013-D - XI Base, Unfors RaySafe, Inc., Billdal, Sweden). The Unfors detector and multimeter are calibrated annually by Unfors RaySafe Inc., with a certified tolerance of 0.3% X-ray dose at 70 kVp.

An anthropomorphic tissue-equivalent phantom (ATOM Max Dental Phantom, Model 711-HN, CIRS Inc., Norfolk, VA) was used as a scattering target for the stray radiation. It should be noted that the ATOM Max Dental phantom is specified for use at 50 keV–25 MeV, while the X-ray energy spectrum from the HIDXDs would include energies below 50 keV. Stray radiation air kerma measurements were made with an 1800 cc ionization chamber (Model 10 × 6–1800, Radcal Corporation, Monrovia, CA) connected to an Accu-Pro electrometer (Model 11251, Radcal Corporation, Monrovia, CA). The large volume of the 1800 cc ionization chamber is ideally suited for measuring the air kerma of low fluence stray radiation, such as for scatter fields, as opposed to smaller volume chambers such as a 0.6 cm^3^ farmer chamber. The ionization chamber specifications met the required capabilities of accurately measuring very low air kerma levels (nGy range) with an appropriately short response time of no greater than 1 ms for the exposure times used with the HIDXDs. The ionization chamber and electrometer pair is calibrated annually by the Measurements Science and Standards Division of the National Research Council (Ottawa, Ontario) and a calibration factor of 9.69 µGy/mR (meter) at 30 kVp was applied to all measurements. The calibration factor for 30 kVp was deemed appropriate based on lab measurements of stray radiation energy, and as used in similar work.^15^ For each measurement at a given spatial location, the radiation was measured, with the detector in dose accumulate mode, for a period of 10 s and repeated three times. The mean value of the three, 10-s measurements has been reported as the stray radiation air kerma value for a given spatial location. Prior to evaluation of the devices, measurements of background radiation over 10 s were made. The initial background radiation measurement was then subtracted from every stray radiation meter reading.

**Figure 1.**
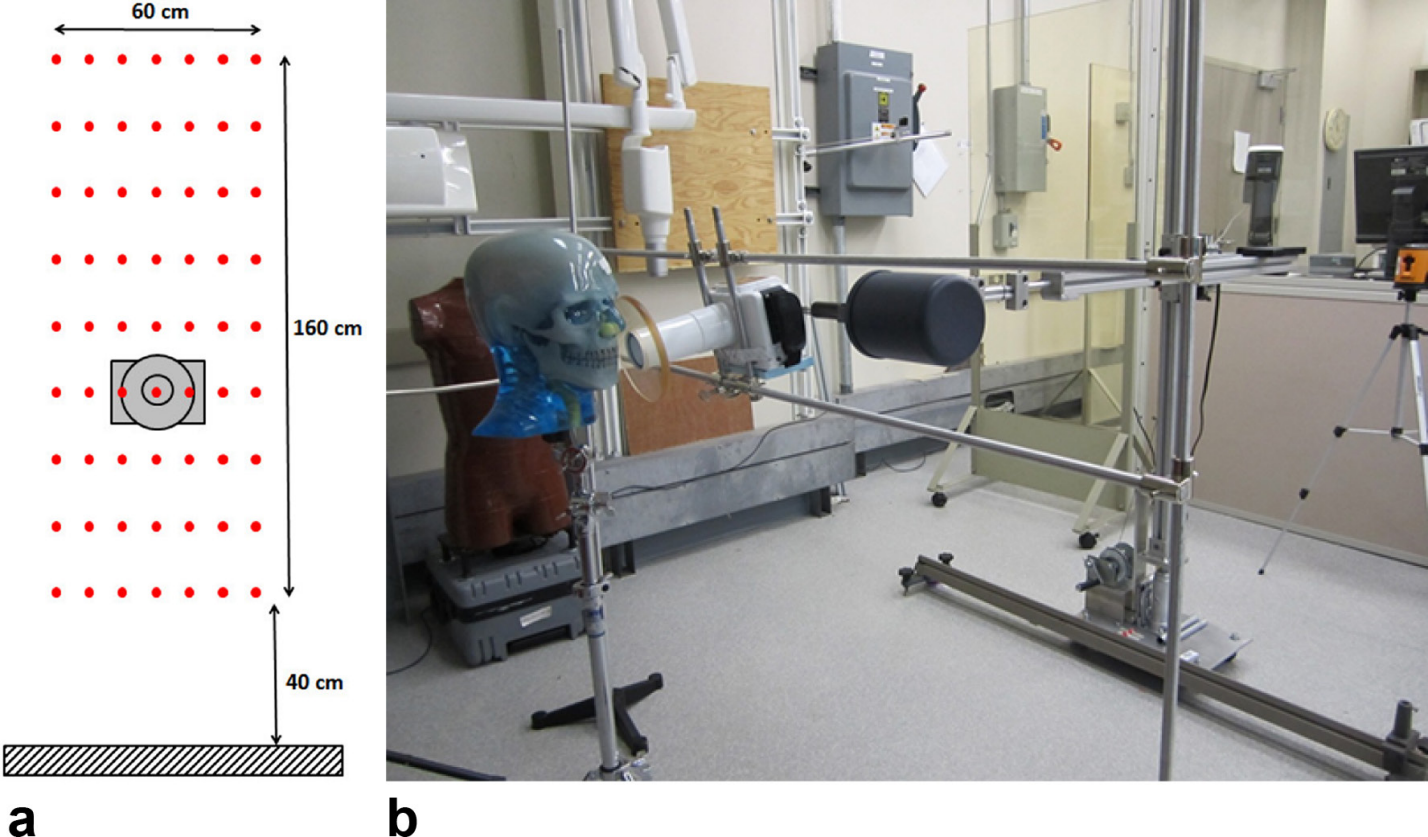
(a) Schematic of the 63 stray radiation air kerma measurement points in a vertical plane 10 cm behind the device located within the significant zone of occupancy for the operator. (b) Picture of the experimental setup for the stray radiation air kerma measurements, consisting of the phantom, hand-held dental X-ray device, and ionization chamber held in place by a custom positioning apparatus.

For each device, measurements at 63 points evenly spaced on a 160 cm high by 60 cm wide grid pattern were taken within a plane located 10 cm behind the device (Figure 1a), as a distance of 10 cm was assumed to be typical for positioning of the HIDXD from the body of the operator. These points are within the significant zone of occupancy, as defined by the International Electrotechnical Commission,^16^ where the operator would typically stand. A custom apparatus was used to securely hold the ionization chamber in place and provide reliable measurement of the spatial location of the chamber (Figure 1b). The HIDXDs were placed on a stand with the distal end of the cone 100 cm vertically from the floor and 2 cm from the phantom. The devices were positioned for a bitewing image of the molars, with the device cone at an angle of 10° below the horizontal plane of the teeth.^17^

For the uncertainty in the stray radiation air kerma measurements, three sources of uncertainty were considered: measurement uncertainty of the ionization chamber and electrometer pair; the stability of the stray radiation field as produced by each individual HIDXD (directly related to the stability of the device output); and the accuracy of the equipment positioning and spatial sampling of stray radiation field. The measurement uncertainty of the meter and stability of the stray radiation field uncertainty attempt to capture the variation from the equipment used. The equipment positioning and spatial sampling uncertainty attempts to capture the variation of the measured stray radiation values due to the positioning of the phantom and ionization chamber within the experimental setup, and the true air kerma value for a given spatial location.

In order to characterize the uncertainty for the equipment positioning and spatial sampling, further measurements were taken. For each of five different spatial locations, including areas of relatively low and high stray radiation air kerma measurements, 10 sets of air kerma measurements were taken at each of those five locations. Each set consisted of three air kerma measurements (x_1_, x_2_, x_3_), with no changes to the experimental setup, yielding a mean value µ_1_ (mean of x_1_, x_2_, x_3_). In between each set at a given location, the ionization chamber and phantom were moved out of position, and then placed back into position for the next set, providing 10 mean values (µ_1,_ µ_2, …_ µ_10_). These 10 mean values at a given spatial location where then used to calculate a value for the estimated uncertainty due to equipment positioning and spatial sampling.

## Results

Table 2 provides a quantitative summary of the measured stray radiation air kerma values in the significantzone of occupancy (SZO) for each device, including the median, mean and maximum measured air kerma values. The standard deviation and interquartile range of the measured air kerma values for each device are also provided to further characterize the distribution of the measurements. A timer setting of 1 s was used for all exposures with each device. As per the variation in air kerma per current–time product and tube currents for the devices indicated in Table 1, setting the timer to 1 s for all measurements means the X-ray output of each device was not equivalent during the stray radiation air kerma measurements.

**Table 2.** Quantitative summary of measured stray radiation air kerma values

Device	Current–time product (mAs)	Median air kerma (nGy)	Mean air kerma (nGy)	Max air kerma (nGy)	Standard deviation	Interquartile range (Q3–Q1)
BIOX-IPX 0	3.0	46	138	438	0.14	234
Nomad Pro2	2.5	124	289	958	0.28	447
SAF-3000	2.0	89	213	666	0.20	340
Xray2Go	2.0	46	138	438	0.14	234
Zen-PX2	2.0	138	182	459	0.14	256

Summary of key quantitative metrics for measured stray radiation air kerma values at all 63 spatial locations within the Significant Zone of Occupancy (SZO). A timer setting of 1.0 sec was used for all device measurements, implying the X-ray output is not constant across the devices. Normalization is required to directly compare the measured stray radiation between devices.

**Figure 2.**
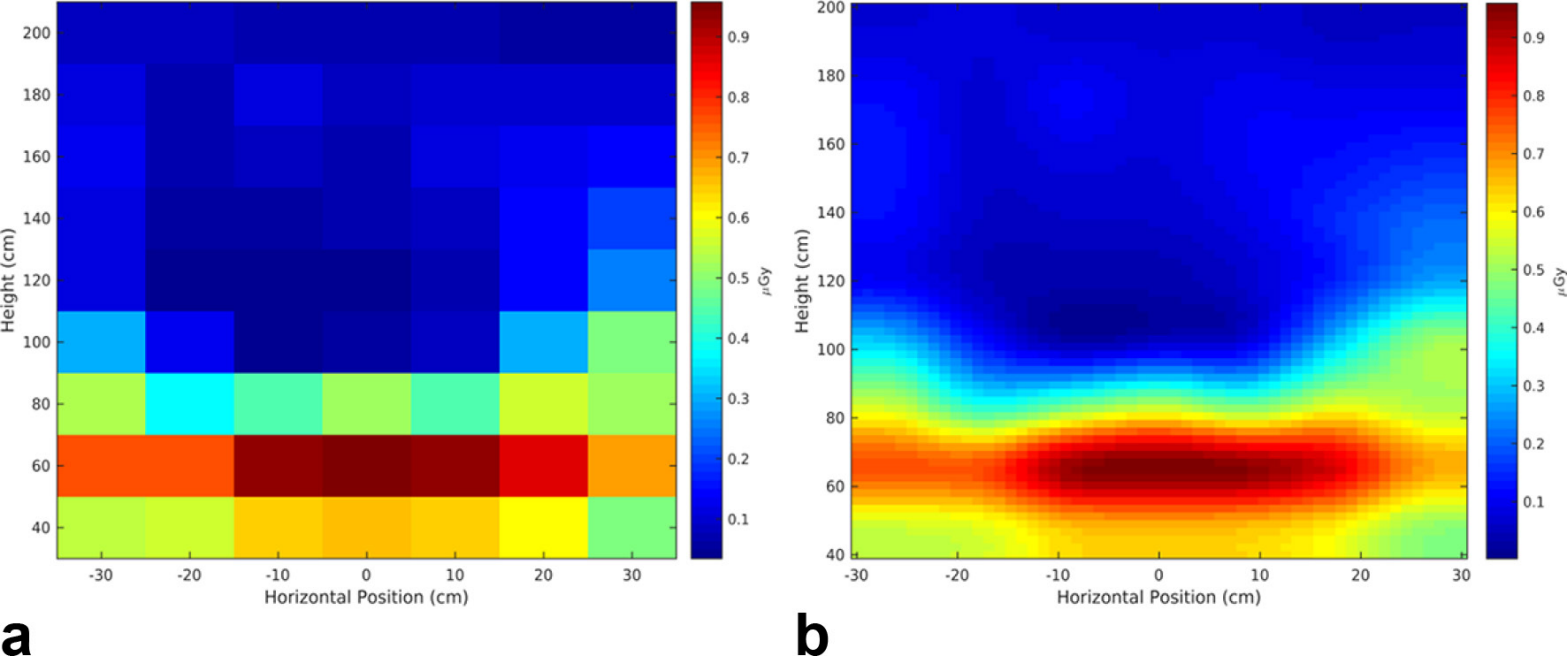
(a) Mapping of the measured stray radiation air kerma values for a hand-held dental X-ray device positioned for a bitewing image of the molars, indicating the spatial variation for each of the measurements. (b) For visual purposes only, a grid of more refined pixels of the measured stray radiation values generated by a bicubic interpolation using a 4 × 4 neighbourhood weighted average (MathWorks, Natick, MA). Note that any calculations used throughout this work were performed with measured values only, and not interpolated values.

Figure 2a provides a characterization of the spatial distribution of the measured stray radiation air kerma values for one of the devices, with each value mapped via colour scale to its representative measurement location. For visual purposes only, Figure 2b provides a smoothed mapping of the measured stray radiation air kerma values by interpolating to a grid with more refined pixels attempting to provide further detail on the heterogeneous nature of the stray radiation field.

The overall uncertainty for each measurement was calculated by adding the relative uncertainties for the three considered sources of uncertainty. The measurement uncertainty of the ionization chamber and electrometer pair was 1% (assuming a confidence level of approximately 95% for a normal distribution) as determined by the Measurements Science and Standards Division of the National Research Council. For the uncertainty in the stability of the stray radiation field as produced by each individual HIDXD, the coefficient of variation (CoV), as calculated for each set of three measurements at a given spatial location, was used to estimate the variability at each measurement location. For measurements at every spatial location and across all HIDXDs, the median CoV was 0.8%. When considering only measurements for a given HIDXD, the largest median CoV was 1.5%. For the measurements taken to characterize the uncertainty due to the accuracy for the equipment positioning and spatial sampling, the CoV for µ_1,_ µ_2, …_ µ_10_ was calculated for each location. This resulted in a range of CoVs from each location of 12–16%. The values added to give the overall estimated uncertainty for each measurement were 1% for the ionization chamber and electrometer pair, 1.5% for the stability of the stray radiation field, and 16% for the accuracy of equipment positioning and spatial positioning. This results in an overall uncertainty of approximately 19% to be applied for each measurement, which represents a conservative approach to estimating the uncertainty of the reported values. A conservative estimate of the overall uncertainty was also deemed to be most appropriate since the ATOM Max Dental Phantom explicitly states that it is for use at 50 keV and higher and the correction to exposure values obtained at energies below 50 keV included in scatter are likely minor and linear in nature.

## Discussion

For the measured stray radiation air kerma values (S_Mes_) for each device, the typical median air kerma values (K_Norm_) required to produce a diagnostically acceptable image on a digital receptor (0.8 mGy) and D-Speed film (1.6 mGy) were used to create a normalized stray radiation air kerma value (S_Norm_). As the stray radiation measurements were all done with a timer setting of 1.0 s for each device, taking the product of the measured air kerma per current–time product (Table 1) with the tube current for a given device (Table 1) provides a calculated output air kerma of each device (K_Calc_). The normalized stray radiation air kerma (S_Norm_) values can then be estimated by Formula (1).



**Figure 3.**
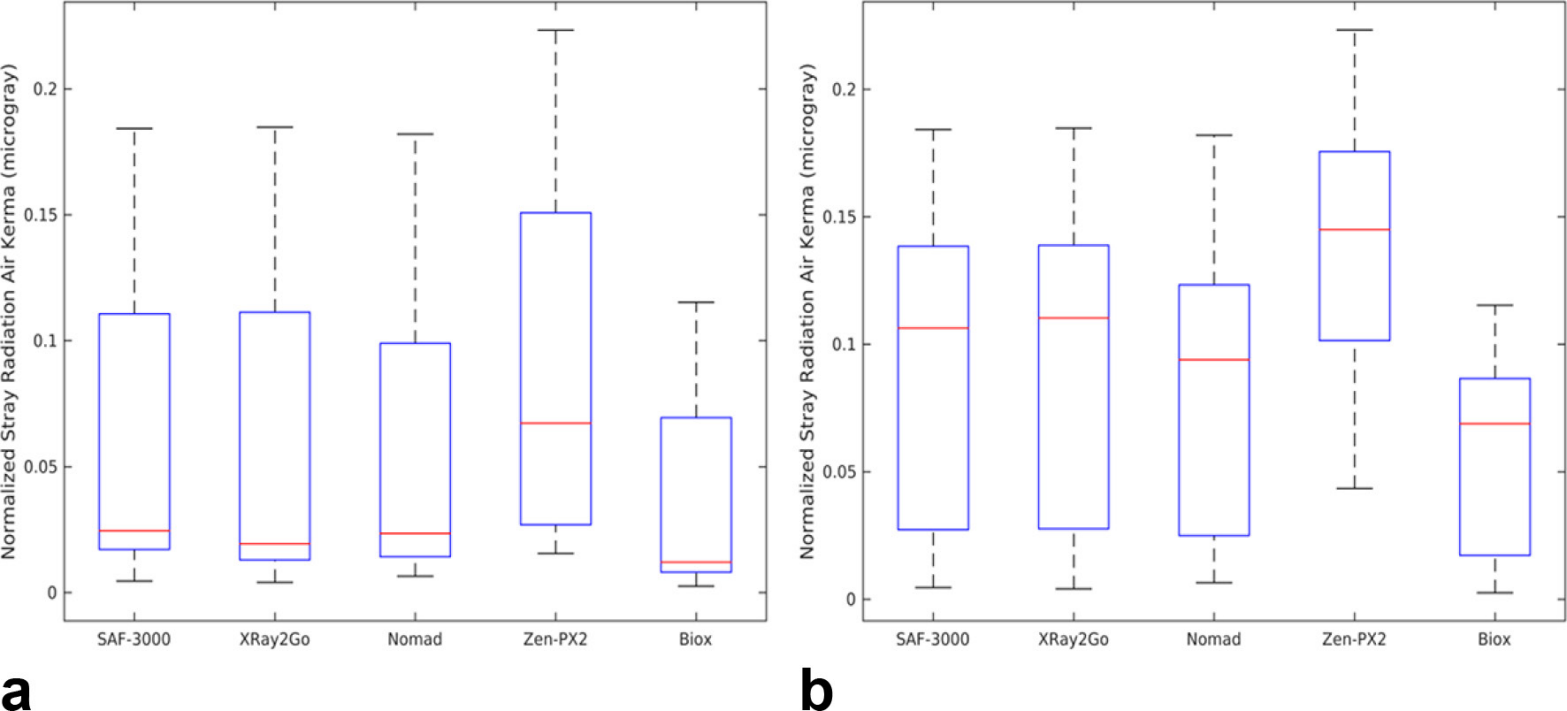
(a) “Box-and-whisker” plots for all the measured stray radiation air kerma values for each HIDXD, as scaled for typical median air kerma output required for digital receptors of 0.8 mGy. (b) The same data sources with only the measured values located at 120 cm and lower included. HIDXD, hand-held intraoral dental X-ray device.

Table 3 provides a quantitative summary of the normalized stray radiation air kerma values (S_Norm_) as calculated for both digital radiographs and D-Speed film. This normalization was done to allow for a fair comparison of the measured stray radiation air kerma values across all HIDXDs, to reflect the differing exposure times that would be required from each device to produce sufficient exposure of a given image receptor. Figure 3a provides a box-and-whisker style plot highlighting the median value, interquartile range, and outlier limits (set at one and a half times the interquartile range) for all five HIDXDs normalized to the median air kerma target values typically required for digital intra oral radiographs. This shows close agreement of the stray radiation fields between the devices, with some differences apparent, presumably due in part to differing filtration among the devices creating variations in beam hardness. Differences in lead equivalence of the backscatter shield for each device would also presumably affect the measured scatter values between devices. Figure 3b provides the same graphical representations as in Figure 3a, except that only measurements located at a height of 120 cm or lower were used to generate the figure, thus focusing on the area of relatively higher measured stray radiation field as indicated in Figure 2.

**Table 3.** Quantitative summary of normalized stray radiation air kerma values (S_Norm_)

Device	Median air kerma (nGy)	Mean air kerma (nGy)	Max air kerma (nGy)
*Median air kerma typically required for D-speed film (1.6 mGy air kerma at end of dental cone*)			
BIOX-IPX 0	28	83	264
Nomad Pro2	54	126	416
SAF-3000	56	135	421
Xray2Go	44	133	423
Zen-PX2	154	203	510
Mean	67	136	407
*Median air kerma typically required for digital receptor (0.8 mGy air kerma at end of dental cone*)			
BIOX-IPX 0	14	41	132
Nomad Pro2	27	63	208
SAF-3000	28	67	211
Xray2Go	22	66	211
Zen-PX2	77	101	255
Mean	34	68	203

Measured stray radiation air kerma values as normalized to the median air kerma values typically required to produce a diagnostically acceptable image on a digital receptor (0.8 mGy) and D-Speed film (1.6 mGy).

As indicated in Figure 2, the stray radiation field varies in the vertical plane of the SZO. The maximum values are observed in the area below 120 cm vertically, which follows from the direction of the primary beam due to its angle of 10° below horizontal. Figure 3 further illustrates the higher measured air kerma values occurring below 120 cm, as the median and interquartile ranges are all shifted up to higher air kerma values. As demonstrated by the difference in the median and mean air kerma values in Table 2, the localized area of high air kerma has significantly larger air kerma values than the surrounding lower values, while being a relatively small area within the significant zone of occupancy. It should be noted that the measured variation in the stray radiation field is specific to the bitewing technique used, where the primary beam was at an angle of 10° below the horizontal. Different intraoral X-ray techniques would presumably create spatial differences in the stray radiation field, with potentially different locations for the maximum measured stray radiation values. An operator of a HIDXD may also hold the device at a height other than the experimental setup of 1 m, such as at chest height or underneath the chin, causing an upwards shift of the localized area of high air kerma if the same intraoral technique were used. Over time, with multiple exposures and potentially different orientations of the HIDXD used, the typical cumulative scatter field that an operator would be exposed to would likely be a combined average of the scatter from the many orientations.

For a conventional wall-mounted intraoral dental X-ray device, the typical yearly full-body effective dose to the operator, as calculated through occupational dosimetry, can be expected to be under 0.1 mSv, with 97% of monitored Canadian dental workers having a reported dose of zero in 2016.^18^ When using conventional dental X-ray equipment, the operator is either positioned at a greater distance from the X-ray source (2−3 m) or standing behind a shielded wall. As a result the stray radiation field to which they are exposed is expected to be far more uniform than for HIDXD devices, and the dose recorded from an occupational dosemeter can give an estimate of the full-body effective dose. Table 4 indicates, for each of the HIDXD devices, estimates for the yearly exposure to the operator when the indicated typical optimized settings are used. In calculating the estimated yearly exposures, a workload of 10 000 exposures per year was used, which is equivalent to the NCRP Report No. 145 intraoral medium volume weekly workload over 50 weeks.^19^ It must be noted that the median, mean, and max air kerma exposure values do not represent a measured full body absorbed dose or a calculated effective dose, but instead are indicators of the varied air kerma measurements of stray radiation in the significant zone of occupancy. With the localized region of relatively higher exposure as indicated in Figure 2, and as demonstrated by the differences between the median, mean, and maximum exposure values in Table 4, it is difficult to estimate the full-body effective dose from an HIDXD with a single occupational dosemeter. This demonstrates a limit when using a single dosemeter badge in occupational dosimetry with HIDXDs, as depending on where the dosemeter is placed, and the fact that operators would likely carry out exams with HIDXDs at varying orientations, it could under- or overestimate the full-body effective dose to the operator, as the dosemeter would assume a uniform X-ray exposure over the whole body.

**Table 4.** Estimated annual operator exposures for workload of 10,000 images

Device	From median air kerma (mGy)	From mean air kerma (mGy)	From max air kerma (mGy)
*Median air kerma target for D-speed setting (1.6 mGy air kerma at end of dental cone*)			
BIOX-IPX 0	0.28	0.83	2.64
Nomad Pro2	0.54	1.26	4.16
SAF-3000	0.56	1.35	4.21
Xray2Go	0.44	1.33	4.23
Zen-PX2	1.54	2.03	5.10
Mean	0.67	1.36	4.07
*Median air kerma target for digital setting (0.8 mGy air kerma at end of dental cone*)			
BIOX-IPX 0	0.14	0.41	1.32
Nomad Pro2	0.27	0.63	2.08
SAF-3000	0.28	0.67	2.11
Xray2Go	0.22	0.66	2.11
Zen-PX2	0.77	1.01	2.55
Mean	0.34	0.68	2.03

From the air kerma values of Table 4, it would be expected that an occupational dosemeter placed within the localized region of relatively higher stray radiation exposure from an HIDXD would record a dose above 0.1 mSv, and therefore above the expected dose for conventional wall-mounted devices. While the occupational dosemeter from an HIDXD would not represent a full-body effective dose, the operator could receive an expected increase in effective dose compared to conventional wall-mounted devices, depending on the relative radiation sensitivity of organs within the high stray radiation region, due to an increase in organ doses within the region. From the air kerma values in Table 4, it can be inferred that organ doses within the region of high stray radiation could increase by an order of magnitude or more compared to conventional wall-mounted devices. For example, if an operator was to use an HIDXD by holding the device at chest level or under the chin, radiation sensitive organs such as the breast, salivary glands, and thyroid could be within the localized region of higher stray radiation measured in this study and therefore receiving increased organ doses compared to conventional wall-mounted devices. Other radiation sensitive organs such as the lens of the eye and gonads could also be impacted by the localized region of higher stray radiation depending on the location and orientation of the HIDXD by the operator. An increase in dose to radiation sensitive organs for HIDXDs compared to conventional wall-mounted devices would impact the overall radiation risk to the operator.

## Conclusion

From five HIDXDs, air kerma measurements for numerous spatial positions have demonstrated a heterogeneous stray radiation field with extrapolated annual air kerma ranges for typical optimized digital image receptors of 0.14–0.77 mGy for the median of the spatial measurements, 0.41–1.01 mGy for the mean, and 1.32–2.55 mGy for the maximum. Similarly, for typical optimized D-speed film the extrapolated annual air kerma ranges were 0.28–1.54 mGy for the median, 0.83–2.03 mGy for the mean, and 2.64–5.10 mGy for the maximum. In order to effectively and accurately characterize the scatter field, measurements were taken across 63 evenly sampled positions within a plane, with an appropriate anthropomorphic tissue-equivalent phantom, using a detector properly calibrated for the required beam energy and with an appropriate ionization chamber volume and short response time for dental X-ray devices. Due to the potential for increase in radiation risk to the operator, in order to keep operator doses as low as reasonably achievable HIDXDs should only be used when the use of a portable device on a stand or a wall-mounted device, activated from a protected area (at a distance or behind a barrier), is not reasonably feasible and patient accessibility makes the use of HIDXDs necessary for the required clinical purpose.

## References

[1] Michigan Department of Community Health Dental Intra-oral Patient Exposures. As referenced in National Council on Radiation Protection & Measurements, NCRP Report No. 172 – Reference Levels and Achievable Doses in Medical and Dental Imaging: Recommendations for the United States. NCRP 2012.

[2] Moyal AE Nationwide Evaluation of X-ray Trends (NEXT): Tabulation and Graphical Summary of the 1999 Dental Radiography Survey. Available from: http://c.ymcdn.com/sites/www.crcpd.org/resource/collection/81C6DB13-25B1-4118-8600-9615624818AA/NEXT99Dental.pdf [February 12, 2018].

[3] Berkhout WE, Suomalainen A, Brüllmann D, Jacobs R, Horner K, Stamatakis HC Justification and good practice in using handheld portable dental x-ray equipment: a position paper prepared by the European Academy of DentoMaxilloFacial radiology (EADMFR. Dentomaxillofac Radiol 2015; 44: 20140343. doi: 10.1259/dmfr.20140343 25710118 PMC4628399

[4] Makdissi J, Pawar RR, Johnson B, Chong BS The effects of device position on the operator's radiation dose when using a handheld portable X-ray device. Dentomaxillofac Radiol 2016; 45: 20150245. doi: 10.1259/dmfr.20150245 26764582 PMC4846146

[5] Rottke D, Gohlke L, Schrödel R, Hassfeld S, Schulze D Operator safety during the acquisition of intraoral images with a handheld and portable X-ray device. Dentomaxillofac Radiol 2018; 47: 20160410. doi: 10.1259/dmfr.20160410 29319336 PMC6047632

[6] Hosseini Pooya SM, Hafezi L, Manafi F, Talaeipour AR Assessment of the radiological safety of a Genoray portable dental X-ray unit. Dentomaxillofacial Radiology 2015; 44: 20140255. doi: 10.1259/dmfr.20140255 25343709 PMC4614165

[7] Cho JY, Han WJ The reduction methods of operator's radiation dose for portable dental X-ray machines. Restor Dent Endod 2012; 37: 160–4. doi: 10.5395/rde.2012.37.3.160 23429415 PMC3569401

[8] Pittayapat P, Oliveira-Santos C, Thevissen P, Michielsen K, Bergans N, Willems G, et al Image quality assessment and medical physics evaluation of different portable dental X-ray units. Forensic Sci Int 2010; 201(1-3): 112–7. doi: 10.1016/j.forsciint.2010.04.041 20554135

[9] Danforth RA, Herschaft EE, Leonowich JA Operator exposure to scatter radiation from a portable hand-held dental radiation emitting device (Aribex NOMAD) while making 915 intraoral dental radiographs. J Forensic Sci 2009; 54: 415–21. doi: 10.1111/j.1556-4029.2008.00960.x 19187461

[10] Iwawaki A, Otaka Y, Asami R, Ozawa T, Izawa M, Saka H The study of protection of operators and surrounding workers at the time of using portable intraoral X-ray unit. Leg Med 2018; 33: 66–71. doi: 10.1016/j.legalmed.2018.05.007 29933235

[11] McGiff TJ, Danforth RA, Herschaft EE Maintaining radiation exposures as low as reasonably achievable (ALARA) for dental personnel operating portable hand-held x-ray equipment. Health Phys 2012; 103(2 Suppl 2): S179–S185. doi: 10.1097/HP.0b013e318259fa29 22739973

[12] Gray JE, Bailey ED, Ludlow JB Dental staff doses with handheld dental intraoral X-ray units. Health Phys 2012; 102: 137–42. doi: 10.1097/HP.0b013e318230778a 22217586

[13] Goren AD, Bonvento M, Biernacki J, Colosi DC Radiation exposure with the NOMAD portable X-ray system. Dentomaxillofac Radiol 2008; 37: 109–12. doi: 10.1259/dmfr/33303181 18239038

[14] Hermsen KP, Jaeger SS, Jaeger MA Radiation safety for the NOMAD portable X-ray system in a temporary morgue setting. Journal of Forensic Sciences 2008; 53: 917–21. doi: 10.1111/j.1556-4029.2008.00766.x 18489554

[15] Marshall NW, Faulkner K, Warren H Measured scattered X-ray energy spectra for simulated irradiation geometries in diagnostic radiology. Med Phys 1996; 23: 1271–6. doi: 10.1118/1.597690 8839423

[16] International Electrotechnical Commission. IEC 60601-1-3 Medical electrical equipment – part 1-3: general requirements for basic safety and essential performance – collateral standard: Radiation protection in diagnostic x-ray equipment (2nd EDN.. Subclause 13.4 2008; IEC.

[17] Iannucci JM, Howerton LJ Dental radiography principles and techniques (4th EDN). Saunders 2012.

[18] Health Canada. 2017 Report on occupational radiation exposures in Canada. 2018.

[19] National Council on Radiation Protection & Measurements NRCP report no. 145 – Radiation protection in dentistry. NCRP 2003.

